# Natural (Clinoptilolite) and Synthetic (NaP1) Zeolites in the Adsorption Process for the Removal of Acid Black 1 Dye from Aqueous Solutions

**DOI:** 10.3390/molecules30081677

**Published:** 2025-04-09

**Authors:** Magdalena Pająk, Agnieszka Dzieniszewska, Joanna Kyzioł-Komosińska

**Affiliations:** Institute of Environmental Engineering Polish Academy of Sciences, 34 M. Skłodowska-Curie Street, 41-819 Zabrze, Poland; agnieszka.dzieniszewska@ipispan.edu.pl (A.D.); joanna.komosinska@ipispan.edu.pl (J.K.-K.)

**Keywords:** adsorption, zeolites, clinoptilolite, NaP1, acid dye, isotherms, kinetic

## Abstract

This study investigates the adsorption of Acid Black 1 (ABk 1) dye onto natural clinoptilolite (CLIN) and synthetic NaP1 zeolites under various conditions of adsorbent dose (5, 10, 20 g/L), dye concentration (1–1000 mg/L), and contact time (5–1440 min). The adsorption data were analyzed using Freundlich and Langmuir isotherms, as well as pseudo-first-order and pseudo-second-order kinetic models. Both linear and nonlinear regression methods were applied to assess the model fit. The results showed that CLIN exhibited maximum adsorption capacities of 35.32 mg/g, 21.9 mg/g, and 9.39 mg/g at doses of 5 g/L, 10 g/L, and 20 g/L, respectively. For NaP1, the corresponding values were 28.44 mg/g, 12.46 mg/g, and 9.11 mg/g. The pseudo-first-order model described adsorption at low dye concentrations and short contact times, while the pseudo-second-order model successfully explained adsorption across all tested conditions. These findings suggest that both zeolites, particularly CLIN, are effective adsorbents for ABk 1 dye removal, presenting a valuable solution for wastewater treatment applications.

## 1. Introduction

Water is a crucial natural resource, indispensable for sustaining life on Earth. One of the greatest challenges facing humanity today is water pollution, which affects ecosystems, public health, and sustainable development. Water pollution arises from industrial operations, agricultural practices, and domestic activities, and it manifests in numerous forms. Among these, synthetic dye pollution is particularly concerning due to the extensive use of dyes in modern industries and their harmful impact on aquatic environments. These pollutants not only degrade water quality but also pose significant threats to both ecosystems and human health [1,2,3]. Addressing the issue of water pollution, particularly from synthetic dyes, is therefore an urgent environmental priority requiring innovative and sustainable solutions.

Textile dyes play a key role in the textile industry, adding colors to fabrics, but they negatively impact the environment due to their high chemical stability, large molecular weights, and poor biodegradability. In addition, their widespread use is associated with serious water pollution problems [4,5]. Textile dyes that are not completely removed during the textile manufacturing process can leach into the aquatic environment and have negative effects on the ecosystem and human health [6]. Water contaminated with textile dyes becomes opaque, altering the natural colors of surface and underground waters. In addition, some dyes have toxic properties and can be harmful to aquatic organisms [7,8]. Removing textile dyes from water is a technological and environmental challenge [9]. Traditional methods employed for the treatment of wastewater contaminated with dyes include adsorption on a solid surface, membrane filtration, ion exchange, electrochemical techniques, coagulation and flocculation, reverse osmosis, chemical oxidation, ozonation, and biological treatments, including activated sludge and bacterial action [1,2,3,6,8,9,10,11,12,13,14,15,16]. The choice of the method depends on its efficiency, environmental impact, and, most critically, its economic feasibility. Adsorption has emerged as one of the most promising approaches for removing textile dyes from water and wastewater. This is due to its simplicity in design, high efficiency, ease of application, cost-effectiveness, and environmental compatibility, particularly its biodegradability [4,5,17,18,19,20,21,22,23]. A wide range of adsorbents have been investigated for their potential to remove textile dyes, including clays [2,8,24,25], activated carbon [26], chitosan—a biopolymer derived from natural sources [27,28,29]—and agricultural and industrial by-products like crop residues, slag, and fly ash [30,31,32,33,34].

Despite their demonstrated effectiveness in removing contaminants, many adsorbents face significant limitations that restrict their broader application, particularly in large-scale water treatment processes. These challenges primarily stem from high production costs, limited adsorption capacities, and low regeneration potential, which make them less feasible for sustained industrial use. Consequently, ongoing research is heavily focused on the development of cost-effective, efficient, and reusable adsorbents that can meet the rigorous demands of modern water treatment systems while addressing environmental and economic concerns. This underscores the need for innovative solutions to tackle textile dye pollution sustainably.

Natural adsorbents, such as zeolites and bentonites, have gained particular attention due to their promising properties, including their high cation exchange capacity, large surface area, and natural abundance. These characteristics make them highly effective for the removal of organic compounds from water, positioning them as compelling alternatives to synthetic adsorbents [8,35,36,37]. Among natural zeolites, clinoptilolite stands out due to its cost-effectiveness and industrial importance. Clinoptilolites, chemically represented as Na_6_[(AlO_2_)_6_(SiO_2_)_30_]·24H_2_O or (Na_2_K_2_CaMg)_3_[(AlO_2_)_6_(SiO_2_)_30_]·24H_2_O, are porous aluminosilicates characterized by a robust three-dimensional skeletal structure that facilitates their exceptional adsorption capabilities [3,38,39]. While natural zeolites like clinoptilolite are highly valued, synthetic zeolites have been shown to have equally high adsorption capacities in many cases, making them an equally attractive option for water treatment [40]. Synthetic zeolites can be engineered to have specific structural and chemical characteristics, allowing for optimized performance in removing dyes and other pollutants. One notable source for the synthesis of these materials is coal fly ash, an industrial by-product. Currently, only about 25% of coal fly ash is utilized in civil engineering applications, leaving the majority of this material to be deposited in landfills. This practice not only diminishes the esthetic value of the affected regions but also introduces significant environmental risks, such as soil and groundwater contamination [41,42].

In recent years, there has been a growing emphasis on the rational management and reuse of waste materials like coal fly ash. The synthesis of zeolites, such as NaP1, from coal fly ash is a promising strategy for addressing both waste management and water pollution challenges. The synthetic zeolite NaP1 exhibits excellent adsorption properties, making it a highly effective material for removing textile dyes from wastewater [40,43,44]. This dual purpose approach—recycling industrial by-products to create efficient adsorbents—not only contributes to environmental sustainability but also offers an economically viable solution to the persistent issue of dye pollution in water systems. Such advancements underline the critical role of innovative material science in driving sustainable water treatment practices. Consequently, the primary aim of this research was to assess and compare the efficiency of Acid Black 1 (ABk 1) dye removal using a natural material, clinoptilolite, and a synthetic zeolite, NaP1.

To ensure the reliability and accuracy of the results, adsorption experiments were conducted using model solutions of the ABk 1 dye rather than real wastewater, avoiding errors from complex wastewater matrices. This approach allowed precise control over experimental parameters and focused on specific variables affecting adsorbent properties. Industrial wastewater often contains auxiliary substances used during the dyeing process, such as salts. These substances can influence adsorption capacity, affecting surface charge and dye properties (ionic nature, hydrophobicity, and solubility), which could skew the results. Thus, controlled model solutions were used to ensure accurate measurements.

The experimental analysis evaluated the ability of clinoptilolite and NaP1 to remove ABk 1 dye under various conditions, including different initial dye concentrations, adsorbent doses, and contact times between the adsorbent and the dye solution. To gain deeper insight into the adsorption mechanisms, the experimental data were analyzed using several isothermal models, such as the Freundlich, Langmuir, and Sips models. These models provided critical information about the adsorption behavior of the dye on the zeolite surfaces and enabled the estimation of maximum adsorption capacities as well as the underlying ion binding mechanisms. In addition to equilibrium modeling, the adsorption kinetics were investigated to elucidate the rate and mechanism of dye removal. Two kinetic equations—pseudo-first-order and pseudo-second-order models—were applied to the experimental data. These models helped characterize the dynamic interaction between the dye molecules and the adsorbent surfaces, offering valuable insights into the potential efficiency and applicability of the tested materials in real-world water treatment scenarios. The comprehensive approach employed in this study highlights the importance of integrating equilibrium, kinetic, and parametric analyses to optimize the adsorbent performance and advance the field of wastewater treatment.

## 2. Results

### 2.1. Characteristic of Adsorbents

The synthesis of NaP1 involved a hydrothermal process, where fly ash was treated with NaOH under atmospheric pressure. Specifically, 20 kg of fly ash was combined with 12 kg of NaOH and 90 dm^3^ of water, with a reaction duration of 24 h at a temperature of 80 °C [45,46]. CLIN was ground and dried at 105 °C until a constant mass was achieved.

**Figure 1:**
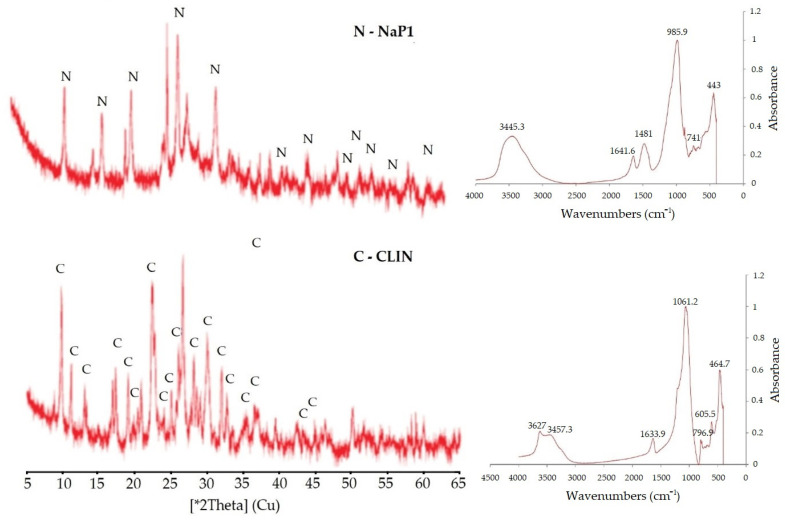
The X-ray patterns and FTiR of the zeolites (*2θ refers to the diffraction angle measured using Cu Kα radiation).

The X-ray analysis results revealed that the tested zeolites were predominantly monomineral (Figure 1). In addition to the zeolite phase, the NaP1 zeolite contained trace amounts of quartz, mullite, and aluminosilicate glass, which is a component of fly ash. For the natural zeolite, the mineral composition was further enriched with small quantities of opal, quartz, feldspars, and mica. The chemical composition of the zeolites was consistent with their mineral composition, with SiO_2_ and Al_2_O_3_ as the predominant components, alongside relatively high levels of calcium and iron oxides. From the FTIR spectra of zeolites, Si-O(Si) and Si-O(Al) bonds were identified in tetrahedra and in aluminum– and silicon–oxygen bridges within the range of 1250–450 cm^−1^. The 800–400 cm^−1^ range corresponds to vibrations characteristic of the zeolite phase, while the 1600–3700 cm^−1^ range is associated with hydroxyl groups and water present in the zeolite structure. Bands below 400 cm^−1^ are attributed to lattice vibrations, indicating the long-range structural order of the framework (Figure 1).

**Figure 2:**
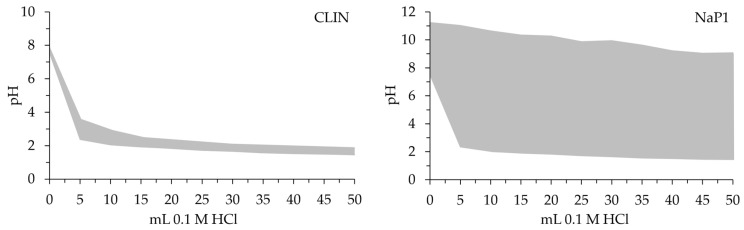
Buffering capacities of CLIN and NaP1.

CLIN and NaP1 differ significantly in their physicochemical properties, which directly influence their adsorption capacities (Table 1). NaP1 exhibits a much higher specific surface area (86.85 m^2^/g) and total pore area (80.236 m^3^/g) compared to CLIN (18.33 m^2^/g and 11.15 m^3^/g, respectively). Additionally, NaP1 has a greater micropore surface (32.84 m^2^/g vs. 10.65 m^2^/g) and porosity (75.33% vs. 54.76%), making it more suitable for adsorption due to the increased availability of adsorption sites. However, CLIN has a larger average pore diameter (0.210 µm vs. 0.070 µm), which may facilitate the adsorption of larger molecules. Another critical factor is the cation exchange capacity (CEC), where NaP1 (138.7 cmol(+)/kg) significantly outperforms CLIN (68.98 cmol(+)/kg), allowing for greater ion exchange during the adsorption process. Moreover, NaP1 exhibited significantly superior buffering properties compared to natural zeolite (Figure 2). The pH of NaP1 is more alkaline (11.22) compared to the nearly neutral pH of CLIN (7.84), which, combined with NaP1’s higher point of zero charge (pH_PZC_ = 8.5 vs. 6.0), indicates that NaP1 remains positively charged over a wider pH range, favoring the adsorption of negatively charged species [18].

In terms of chemical composition, CLIN contains a higher SiO_2_ content (73.9%) and a higher Si/Al ratio (4.79), making it more hydrophobic and less effective for ion exchange. In contrast, NaP1’s lower SiO_2_ content (44.3%) and higher Al_2_O_3_ content (29.0%) result in a lower Si/Al ratio (1.34), which enhances its ion exchange capabilities and affinity for polar or ionic adsorbates. Overall, the higher surface area, porosity, cation exchange capacity, and favorable chemical and pH properties of NaP1 may suggest that it would be a superior adsorbent compared to clinoptilolite, especially for applications involving polar or charged molecules.

### 2.2. Adsorption of ABk 1 onto CLIN and NaP1

**Figure 3:**
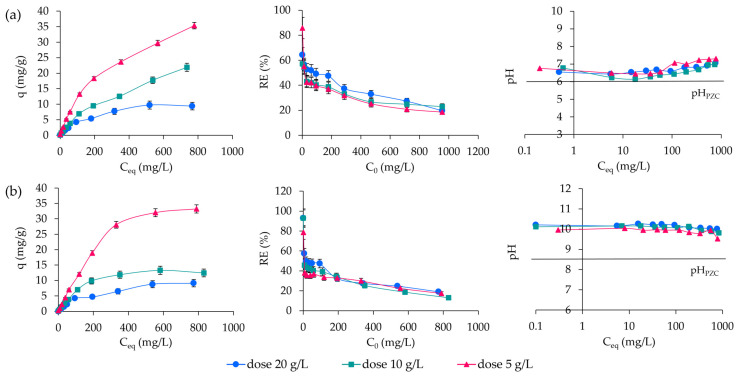
Adsorption capacity (q), removal efficacy (RE), and changes in equilibrium solution pH during adsorption of ABk 1 on CLIN (**a**) and NaP1 (**b**).

The adsorption capacity (q) was analyzed in relation to the equilibrium concentration (C_eq_), while the removal efficiency (RE) was evaluated to illustrate the percentage of ABk 1 removed from aqueous solutions, based on the initial concentration (C_0_) (Figure 3).

The adsorption capacities of CLIN and NaP1 for ABk 1 dye were evaluated at varying adsorbent dosages. The adsorption capacities at the maximum initial concentration observed for CLIN were 35.32 mg/g (RE = 18.5%), 21.9 mg/g (RE = 22.9%), and 9.39 mg/g (19.7%) for adsorbent doses of 5 g/L, 10 g/L, and 20 g/L, respectively. While for NaP1, the corresponding adsorption capacities were 28.44 mg/g (RE = 14.9%) for an adsorbent dose of 5 g/L, 12.46 mg/g (RE = 13.04%) for 10 g/L, and 9.11 mg/g (RE = 19.07%) for 20 g/L. The variation in the adsorption capacity with different adsorbent doses can be attributed to the availability of active sites on the adsorbents and their utilization efficiency. At lower adsorbent doses (5 g/L), the dye molecules have greater access to a larger proportion of the available adsorption sites, resulting in higher adsorption capacities (mg/g). However, as the adsorbent dose increases, the number of available adsorption sites also increases, but the amount of dye adsorbed per unit mass of the adsorbent decreases due to the saturation of the dye in the solution. This effect is particularly evident in the higher doses (10 g/L and 20 g/L), where the adsorption capacity diminishes as the ratio of available dye to adsorbent mass decreases [3].

The adsorption of ABk 1 onto both CLIN and NaP1 occurred at pH levels higher than their respective points of zero charge (pH_PZC_). pH_PZC_ is a critical parameter that defines the pH at which the surface charge of the adsorbent is neutral. At pH levels above pH_PZC_, the surface of the adsorbent becomes negatively charged, which facilitates the electrostatic attraction of cationic or positively charged dye species. In the case of ABk 1, the dye exists in anionic forms in solution. Despite this, the adsorption mechanism may involve non-electrostatic interactions, such as van der Waals forces or hydrogen bonding, which dominate under these conditions. Moreover, the pH-dependent behavior confirms that the adsorbent surfaces remain in a deprotonated state, improving their ability to interact with the dye molecules. The higher adsorption capacities observed at lower adsorbent doses (5 g/L) further underline the importance of these interactions, as the lower surface coverage allows the adsorbents to better exploit their active sites for dye removal. Similar conclusions were drawn by Bień et al. [40] based on their studies on the ability of NaP1 zeolite to remove anionic dye.

Despite the significantly higher specific surface area (86.85 m^2^/g) and porosity (75.33%) of the synthetic zeolite NaP1 compared to CLIN (18.33 m^2^/g and 54.76%, respectively), CLIN demonstrated a superior adsorption capacity for ABk 1. This outcome can be attributed to several key factors. First, CLIN has a larger average pore diameter (0.210 µm) compared to NaP1 (0.070 µm), making its pores more accessible to the relatively large ABk 1 dye molecules. In contrast, the smaller pores of NaP1 likely hinder the diffusion and adsorption of the dye, despite its higher overall porosity and pore volume. Secondly, the higher Si/Al ratio of CLIN (4.79 vs. 1.34 for NaP1) reduces the density of negative charges on its surface, minimizing competition between dye molecules and cations in solution for adsorption sites. Additionally, the greater SiO_2_ content of CLIN (73.9%) contributes to a more hydrophobic surface, enhancing the adsorption of organic dye molecules. Furthermore, the adsorption behavior was influenced by the pH and pH_PZC_ of the materials. CLIN, with a pH_PZC_ of 6.0 and a pH of 7.84, provided a more negatively charged surface under experimental conditions compared to NaP1 (pH_PZC_ = 8.5, pH = 11.22). This enhanced the electrostatic interactions with the anionic dye molecules, promoting stronger adsorption onto CLIN.

The tested zeolites proved to be effective adsorbents for the dye ABk1. Table 2 shows the results of comparing the adsorption capacities of other zeolite adsorbents against various synthetic dyes.

### 2.3. Adsorption Isotherms

**Figure 4:**
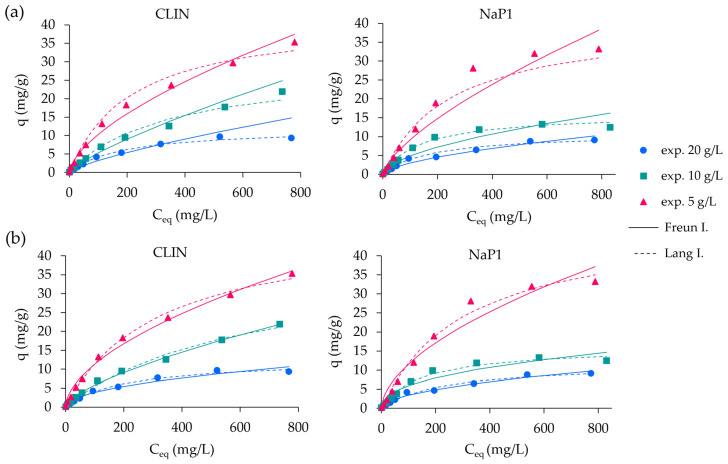
Comparison of experimental data with adsorption isotherms of ABk 1 onto CLIN and NaP1 ((**a**)—linear; (**b**)—nonlinear).

The equilibrium adsorption of ABk 1 onto CLIN and NaP1 was analyzed using two widely applied isotherm models: Freundlich and Langmuir. These models provided parameters that offer valuable insights into the adsorption capacity, surface heterogeneity, and adsorption energy. The parameters were determined through linear and nonlinear regression analyses, with their corresponding coefficients of determination (R^2^) and error metrics (sum of squared errors (SSEs), root mean squared error (RMSE), and χ^2^) presented in Table 3 and Table 4. The theoretical isotherms, calculated using the parameters derived from both linear and nonlinear regressions, were plotted alongside the experimental data and are presented in Figure 4.

For both regression methods, the Freundlich model provided strong fits to the experimental data, as indicated by its high R^2^ values. Nonlinear regression generally yielded slightly better fits (R^2^ values up to 0.9967 for CLIN and 0.9763 for NaP1). The adsorption intensity parameter (1/n) was consistently lower for NaP1, suggesting stronger adsorption favorability, particularly at lower adsorbent dosages. This was complemented by higher K_F_ values for NaP1 in nonlinear regression (e.g., 0.8241–0.8607), demonstrating its superior adsorption capacity relative to CLIN. Linear regression produced lower K_F_ values, but the trends across adsorbent dosages were consistent, with higher adsorption intensities observed at reduced dosages. Nonlinear regression for the Freundlich model showed lower error values (SSE, χ^2^, RMSE) for CLIN and NaP1, although NaP1 had higher error values at lower dosages due to challenges in capturing heterogeneity. Despite this, nonlinear regression was more reliable than linear regression in estimating the adsorption capacity and intensity. The high R^2^ values obtained for the Freundlich model in this study align with findings from Shankar et al. [3], who also observed a strong fit for natural adsorbents, with the Freundlich model effectively capturing surface heterogeneity. The lower 1/n values for NaP1 suggest that it exhibits a stronger affinity for ABk 1, which corroborates the observations of Alpat et al. [4], who noted that Na-X zeolites tend to outperform other zeolites in terms of adsorption at lower adsorbent dosages. The nonlinear regression analysis, which yielded better fits than linear regression, is consistent with findings from Gollakota et al. [37] and Bień et al. [40], who highlighted the superiority of nonlinear methods for accurate adsorption modeling.

The Langmuir model parameters, derived from both regression methods, revealed that NaP1 consistently achieved higher maximum adsorption capacities (Q), particularly under nonlinear regression where Q reached 48.92 mg/g at the lowest dosage. In contrast, Q for CLIN peaked at 48.17 mg/g. Linear regression underestimated Q for both zeolites compared to nonlinear regression. The Langmuir constant (K_L_), reflecting the affinity of the binding sites, was generally higher for NaP1, reinforcing its enhanced adsorption efficiency. The dimensionless separation factor (R_L_) consistently indicated favorable adsorption for both zeolites (0 < R_L_ < 1), with slightly more favorable values observed for NaP1. The favorable R_L_ values observed in both zeolites align with the literature, suggesting efficient adsorption processes, as reported by Babu and Murthy [10] and Pająk and Dzieniszewska [23]. Nonlinear regression provided significantly better fits to the Langmuir model, with R^2^ values ranging from 0.9817 to 0.9957 for NaP1 and 0.9908 to 0.9957 for CLIN. Additionally, error functions such as the SSE, the RMSE, and χ^2^ confirmed the superiority of nonlinear regression in capturing adsorption behavior, with lower error values across all conditions.

### 2.4. Adsorption Kinetics

**Figure 5:**
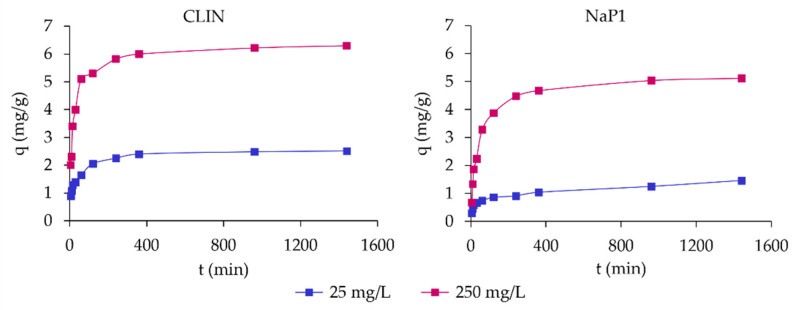
Adsorption kinetic of ABk1 on CLIN and NaP1.

The obtained data were modeled using two pseudo-first-order and pseudo-second-order kinetic models. Table 5 presents the parameters of the two kinetic models of ABk 1 adsorption on CLIN and NaP1, while Figure 5 presents adsorption the kinetics of ABk1 dye on CLIN and NaP1.

The pseudo-first-order model, which assumes that the rate of adsorption is proportional to the number of unoccupied sites, yielded limited accuracy in describing the experimental data for both CLIN and NaP1. For CLIN, the equilibrium adsorption capacity (q_e1_) values were 1.2159 mg/g and 2.6797 mg/g for initial dye concentrations of 25 mg/L and 250 mg/L, respectively. The rate constants (k_1_) were similar, at 0.0044 and 0.0042 min^−1^, and the correlation coefficients (R^2^) were 0.8820 and 0.8270, suggesting only a moderate fit. Similarly, NaP1 exhibited q_e1_ values of 0.9408 mg/g and 3.0826 mg/g for 25 mg/L and 250 mg/L, respectively, with rate constants of 0.0017 min^−1^ and 0.0042 min^−1^. The R^2^ values, 0.8461 and 0.9113, indicated that the pseudo-first-order model inadequately represented the adsorption behavior. The moderate fit of this model suggests that physical adsorption processes alone cannot explain the observed kinetics.

In contrast, the pseudo-second-order model provided a much more accurate representation of the adsorption data, indicating that chemisorption plays a significant role. This model assumes that the adsorption rate depends on the availability of both the adsorbate and adsorbent active sites. For CLIN, the predicted equilibrium adsorption capacities (q_e2_) were 2.5484 mg/g and 6.3573 mg/g for 25 mg/L and 250 mg/L, respectively. The rate constants (k_2_) were 0.0457 and 0.0568 g/mg·min, and the correlation coefficients (R^2^) were nearly perfect, at 0.9997 and 0.9999. For NaP1, the q_e2_ values were slightly lower, at 1.4384 mg/g and 5.2356 mg/g for 25 mg/L and 250 mg/L, respectively. The rate constants (k_2_) were 0.0157 and 0.0277 g/mg·min, and the R^2^ values were similarly high, at 0.9855 and 0.9999. The superior fit of the pseudo-second-order model for both zeolites underscores the dominance of chemisorption mechanisms, likely involving chemical bonding, electron sharing, or exchange interactions. The comparison of q_e_ and q_e2_ values for ABk1 adsorption on CLIN and NaP1 suggests that the pseudo-second-order model provides a more accurate description of the adsorption process. The q_e2_ values are very close to the experimentally determined q_e_, whereas the pseudo-first-order model (q_e1_) significantly underestimates the adsorption capacity, especially at higher concentrations.

When comparing the two materials, CLIN demonstrated a higher adsorption capacity (q_e2_) than NaP1, particularly at a higher dye concentration of 250 mg/L, where q_e2_ reached 6.3573 mg/g for CLIN and 5.2356 mg/g for NaP1. This indicates that CLIN may possess a larger number of active sites or stronger chemical interactions with the dye molecules. Additionally, the rate constants (k_2_) for CLIN were generally higher, reflecting faster adsorption kinetics relative to NaP1. The nearly perfect R^2^ values for both materials confirm that the pseudo-second-order model is highly suitable for describing the adsorption process. According to Shankar et al. [3], the adsorption process on natural zeolites like CLIN follows the pseudo-second-order model, which aligns with our results where both CLIN and NaP1 exhibited high R^2^ values, indicating a chemical adsorption mechanism. This suggests that adsorption on CLIN occurs more rapidly, as shown by higher rate constants (k_2_), compared to NaP1, which can be attributed to the more accessible and effective adsorption sites on CLIN. This is consistent with findings by Gollakota et al. [37], who noted faster adsorption on zeolites with higher surface porosity, such as CLIN. In contrast, NaP1 demonstrated more surface heterogeneity, which may explain its slower adsorption rate and lower adsorption capacity at higher dye concentrations, as discussed by Cheng et al. [43]. Furthermore, the high values of k_2_ for CLIN, as observed in our study, support the idea that its adsorption sites are more readily available for dye molecules, leading to more efficient adsorption compared to NaP1. These results corroborate the work of Alpat et al. [4], who highlighted the influence of the adsorbent structure on the adsorption rate.

## 3. Materials and Methods

### 3.1. Adsorbents—Zeolites

**Figure 6:**
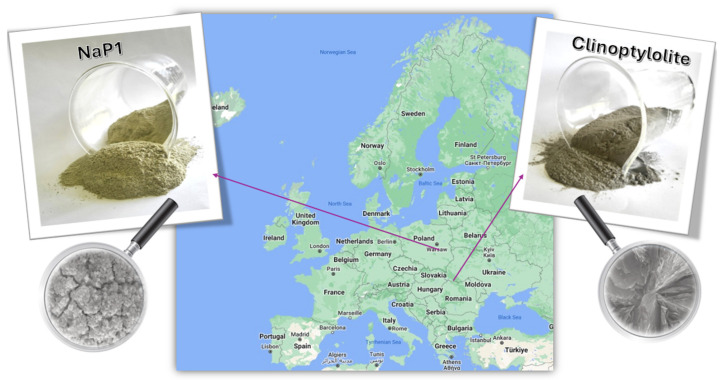
Location of Sokornica deposit (CLIN) and Kozienice power station (NaP1) [51].

In laboratory experiments, two types of zeolites were employed as adsorbents: natural clinoptilolite (CLIN) and synthetic NaP1 (Figure 6). The natural clinoptilolite zeolite was sourced from the Sokornica deposit in Ukraine, while the synthetic NaP1 was produced from fly ash obtained from the combustion of hard coal at the “Kozienice” power plant (ENEA Kozienice Polska Power Plant). The natural clinoptilolite used in this study had a grain size of below 0.2 mm, while the synthetic NaP1 zeolite was finer, with a grain size of below 0.15 mm.

The selected zeolites were characterized in terms of their mineral and chemical composition, specific surface area, pore size distribution, cation exchange capacity (CEC), buffer capacity, surface functional groups, and pH.

The chemical composition was determined via X-ray fluorescence spectroscopy, using a ZSX PRIMUS II spectrometer (Rigaku, Tokyo, Japan). A 20% addition of potato starch was used under a pressure of 20 tons. The mineral composition was analyzed through powder X-ray diffraction (XRD) within the angular range of 5 to 65 2θ, employing a Philips X’pert APD diffractometer equipped with a PW 3020 goniometer, a Cu lamp, and a graphite monochromator (Philips, Eindhoven, The Netherlands). Mineral phase identification was performed based on the ICDD PDF-2 database (2010 edition).

The porous structure of the zeolites was analyzed by nitrogen adsorption/desorption at 77 K using a Micromeritics ASAP 2020 surface area and porosity analyzer (Micromeritics Instrument Corp., Norcross, GA, USA). The specific surface area (SSA) was calculated according to the Brunauer–Emmett–Teller (BET) theory for multilayer adsorption, within the range of p/p_0_ = 0.06–0.3, where p is the equilibrium pressure and p_0_ is the saturated nitrogen vapor pressure. Data analysis was conducted using ASAP 2020 software.

The total porosity was measured using a Carlo Erba mercury porosimeter, model 2000 (Carlo Erba Instruments, Milan, Italy), which applies a maximum pressure of 200 MPa and measures pores within the size range of 10.0 to 3.8 × 10^−3^ μm.

The cation exchange capacity (CEC) was determined by quantifying exchangeable cations (Ca^2+^, Mg^2+^, K^+^, Na^+^) extracted in a 1 M ammonium acetate solution at a pH of 7. The buffer capacity was evaluated using the method described by Jansen, which calculates the area between the pH curves of water suspensions of the samples after the addition of varying amounts of 0.1 M HCl solution, compared to a control curve for quartz sand, which lacks buffering properties [52].

The pH values of the zeolites were measured in deionized water at a 1:2.5 suspension ratio using a pH meter equipped with a combination electrode (glass membrane and reference electrode, ERH-111 Hydromet, Gliwice, Poland). The point of zero charge (pH_PZC_) was determined using the method outlined by Calvete et al. [26], which identifies the pH at which the charge of colloidal particles equals zero.

### 3.2. Adsorbate—Dye

**Figure 7:**
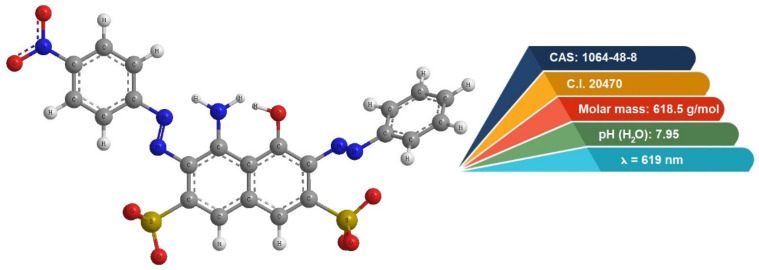
The characteristics of ABk 1 dye.

The anionic dye Acid Black 1 (ABk 1) was selected for adsorption studies. It is a textile diazo dye that is commonly used for dyeing textiles, wool, nylon, and silk, and is also utilized for printing on textiles, foodstuffs, medicines, and cosmetics. Its chemical structure, its properties, and the wavelengths at which the absorbance (λ) was measured are shown in Figure 7. The dye was produced by Boruta Zachem-Kolor, Ltd. (Bydgoszcz, Poland).

Acid Black 1, denoted as ABk 1 and commonly known as Amido Black Staining Solution 2X, Naphthol Blue Black solution, Amido Black 10B, or Buffalo Black NBR, is an example of an azo dye.

### 3.3. Adsorption Experiments

The adsorption of ABk 1 dye onto two types of zeolites, CLIN and NaP1, was investigated through batch experiments conducted at room temperature (23 ± 2 °C). The zeolite–dye suspensions were prepared by adding a known amount of the adsorbent (2 g, 1 g, or 0.5 g) to 100 mL of the dye solution. These suspensions were then subjected to agitation on a horizontal shaker (180 rpm) for a duration of 24 h to ensure that the adsorption reached equilibrium. Once equilibrium was achieved, the solid phase (adsorbent) was separated from the solution by filtration. A stock solution of ABk 1 dye with a concentration of 1000 mg/L was initially prepared by dissolving the bulk form of the dye in double-distilled water. This stock solution was subsequently diluted to produce a series of working solutions with concentrations ranging from 1 to 1000 mg/L, which were used for the adsorption tests.

The adsorption experiments were performed with three different dosages of zeolites, 5 g/L, 10 g/L, and 20 g/L, to evaluate the effect of the adsorbent dose on the removal efficiency. To monitor the adsorption process, the concentrations of ABk 1 dye at both the initial (C_0_ and equilibrium (C_eq_) stages were determined using UV-visible spectrometry, with measurements taken at a wavelength of λ = 619 nm, a characteristic absorbance peak for ABk 1. The pH of the equilibrium solutions was also measured to assess the potential impact of pH changes on the dye removal process.

The amount of adsorbed ABk 1 (q) and the removal efficiency (RE) were calculated using the following equations:

(1) $$q=\frac{\left(C_{0}-C_{eq}\right)\cdot V}{m}$$

(2) $$RE=\frac{\left(C_{0}-C_{eq}\right)}{C_{0}}\cdot 100$$

where C_0_ and C_eq_ are the initial and equilibrium dye concentrations (mg/L), respectively, V is the volume of the ABk 1 solution (L), and m is the mass of the adsorbent (g).

All experimental procedures were carried out in triplicate to ensure the reliability and reproducibility of the results. The data obtained from the experiments were analyzed, and the mean values along with the standard deviations (SDs) were calculated using Microsoft Office 365 software. The standard deviation was used to assess the variation within the experimental results, ensuring the accuracy and consistency of the findings.

### 3.4. Isotherms

Two-parameter adsorption isotherm models—Freundlich and Langmuir—were employed to evaluate the maximum adsorption capacities of CLIN and NaP1, as well as to investigate the mechanisms underlying ABk 1 adsorption. The parameters of these isotherms were calculated using both linear and nonlinear regression methods, as detailed in Table 6. To determine the parameter values in the isotherm equations, nonlinear regression was performed using the classical least squares approach, implemented via the Gauss–Newton algorithm in Statistica software (version 9.0). The quality of fit between the isotherm models and the experimental data were assessed using the coefficient of determination (R^2^), alongside three additional nonlinear error functions: the sum of squared errors (SSEs), the root mean squared error (RMSE), and the chi-square test (χ^2^), following the methodology outlined by Foo and Hameed [53].

### 3.5. Kinetics

Kinetic studies were conducted to examine the adsorption process of ABk 1 dye onto zeolite materials, using initial dye concentrations of 25 mg/L and 250 mg/L, with an adsorbent dose of 10 g/L. The experiments were performed in duplicate to ensure the reliability and reproducibility of the results. Dye concentrations in the solution were measured at specific time intervals, 5, 10, 15, 30, 60, 120, 240, 360, 960, and 1440 min, during which suspensions of NaP1 and CLIN zeolites were subjected to continuous shaking.

To determine the adsorption mechanism and select the kinetic model that best describes the adsorption behavior, two widely used models were applied: the pseudo-first-order and pseudo-second-order kinetic models. The linearized forms of these models, as well as the corresponding equations, are presented in Table 7. The pseudo-first-order model is based on the assumption of proportionality between the rate of adsorption and the difference between equilibrium adsorption and adsorption at a given time, while the pseudo-second-order model considers chemisorption as the rate-limiting step.

## 4. Conclusions

This study presents a novel approach to wastewater treatment by utilizing both natural clinoptilolite (CLIN) and synthetic NaP1 zeolites to effectively remove Acid Black 1 (ABk 1) dye from aqueous solutions. A key highlight of the research is the innovative use of synthetic zeolite derived from fly ash, an industrial waste product. This aligns with contemporary trends in sustainable resource management, emphasizing the reuse of waste materials over disposal. By converting fly ash into NaP1 zeolite, the study not only contributes to waste reduction but also introduces a cost-effective adsorbent with properties that rival those of natural clinoptilolite.

The research demonstrates the efficiency of both CLIN and NaP1 in removing ABk 1 dye across a range of experimental conditions, including different adsorbent doses, dye concentrations, and contact times. While both zeolites proved to be effective, CLIN exhibited a higher maximum adsorption capacity, despite NaP1 having a superior surface area and porosity. This difference is attributed to the unique physicochemical properties of CLIN, such as its larger pore size and higher SiO_2_ content, which enhance its ability to adsorb ABk 1.

The adsorption data were well described by the Freundlich and Langmuir models, with the nonlinear regression method offering the most accurate fits, particularly for NaP1. Kinetic analysis using the pseudo-second-order model confirmed that chemisorption dominates the adsorption process for both zeolites. CLIN, in particular, demonstrated faster adsorption kinetics and a greater capacity at higher dye concentrations, likely due to its more accessible adsorption sites.

Overall, this study provides valuable insights into the adsorption mechanisms of ABk 1 on clinoptilolite and NaP1, with a strong indication that these zeolites—especially CLIN—hold significant potential for wastewater treatment applications, particularly in dye removal.

## Figures and Tables

**Table 1:** Physicochemical properties of zeolites.

Zeolites	CLIN	NaP1
Specific surface area (m^2^/g)	18.33	86.85
Total pore area (m^3^/g)	11.15	80.236
Micropore surface (m^2^/g)	10.65	32.84
Average pore diameter (µm)	0.210	0.070
Porosity (%)	54.76	75.33
CEC (cmol(+)/kg)	68.98	138.7
pH	7.84	11.22
pH_PZC_	6.0	8.5
Chemical composition (%)		
SiO_2_	73.9	44.3
Al_2_O_3_	13.6	29.0
Fe_2_O_3_	2.79	7.81
MnO	0.108	0.125
Na_2_O	0.63	3.97
K_2_O	4.02	1.19
MgO	0.297	0.513
CaO	3.75	10.7
P_2_O_5_	0.222	0.447
TiO_2_	0.296	1.26
Cl	0.0186	0.0186
SO_3_	0.076	0.183
Si/Al, mol·mol^−1^	4.79	1.34

**Table 2:** Comparison of adsorption capacities of zeolite adsorbents in relation to synthetic dyes.

Adsorbent	Dye	Parameters	Adsorption Capacity (mg/g)	References
Na-X	Reactive Black 5	pH = 2, dosage = 0.05/25 mL, T = 25 °C, t = 90 min	q_max_ = 25.3	[1]
	Brilliant Green	pH = 10, dosage = 0.05/25 mL, T = 25 °C, t = 90 min	q_max_ = 24.13	
Clinoptilolite	Violet 5BN	pH = 5, dosage = 1.5 g/L, T = 30 °C, t = 90 min	q_max_ = 7.96	[3]
Clinoptilolite	Reactive Black 5	pH = 6.5, dosage = 0.5 g/100 mL, T = 22.5 °C, t = 10 min	q_max_ = 60.61	[7]
	Reactive Red 239		q_max_ = 111.11	
	Reactive Yellow 176		q_max_ = 88.50	
Na-X	Rhodamine 6G	pH = 2–5, dosage = 0.1–0.5 g/30 mL, C_0_ = 50 mg/L, T = 45 °C	q_max_ = 65.85	[37]
	Alizarin Red S		q_max_ = 76.33	
NaP1	Acid Red 18	pH = 6, dosage = 0.1 g/L, C_0_ = 50 mg/L, T = 23 °C, t = 120 min	q_max_ = 5.39	[40]
NaP1	Methylene Blue	pH = 7, dosage = 1 g/L, C_0_ = 50 mg/L, T = 25 °C, t = 120 min	q_eq_ = 48.7	[43]
Zeolite	Methylene Blue	pH = 6.65, dosage = 1 g/L, C_0_ = 18 mg/L, T = 20 °C, t = 60 min	q_eq_ = 11.21	[47]
Natural zeolite	Methylene Blue	dosage = 0.6 g/L, C_0_ = 20 mg/L, T = 25 °C, t = 120 min	q_eq_ = 1.50	[48]
Synthetic zeolite	Methylene Blue	pH = 6, dosage = 6 g/L, C_0_ = 200 mg/L, T = 40 °C, t = 200 min	q_eq_ = 33.57	[49]
Synthetic zeolites from fly ash	Methylene Blue	dosage = 6.25 g/L, T = 20 °C, t = 60 min	q_eq_ = 5.99	[50]
Clinoptilolite	Acid Black 1	dosage = 5 g/L, C_0_ = 1000 mg/L, T = 23 °C, t = 24 h	q_eq_ = 35.32	This study
NaP1			q_eq_ = 33.20	

**Table 3:** Isotherm parameters—linear regression analysis.

	CLIN			NaP1		
Adsorbent Dosage	20 g/L	10 g/L	5 g/L	20 g/L	10 g/L	5 g/L
Freundlich isotherm						
1/n	0.7490	0.7749	0.6304	0.5899	0.5675	0.6930
K_F_	0.1023	0.1492	0.5630	0.2011	0.3578	0.3749
R^2^	0.9802	0.9925	0.9912	0.9799	0.9596	0.9710
Langmuir isotherm						
q_exp_	9.39	21.90	35.32	9.11	12.46	33.20
Q	11.99	28.41	42.19	10.83	16.08	40.65
K_L_	0.005534	0.003050	0.004637	0.005964	0.007357	0.004001
R_L_	0.1591	0.2556	0.1842	0.1493	0.1246	0.2074
R^2^	0.9845	0.9161	0.9258	0.9390	0.9631	0.8792

K_F_ ((mg/g)·(L/mg)^1/n^), q (mg/g), Q (mg/g), K_L_ (L/mg).

**Table 4:** Isotherm parameters—nonlinear regression analysis.

	CLIN			NaP1		
Adsorbent Dosage	20 g/L	10 g/L	5 g/L	20 g/L	10 g/L	5 g/L
Freundlich isotherm						
1/n	0.4947	0.6632	0.5638	0.5136	0.4284	0.5644
K_F_	0.3977	0.2739	0.8432	0.3206	0.8241	0.8607
R^2^	0.9621	0.9967	0.9924	0.9763	0.9210	0.9549
SSE	4.732	1.715	10.68	2.458	19.43	70.25
*χ* ^2^	1.463	0.4452	1.581	0.9163	3.628	5.870
RMSE	0.6879	0.4142	1.034	0.4958	1.394	2.650
Langmuir isotherm						
q_exp_	9.39	21.90	35.32	9.11	12.46	33.20
Q	12.51	38.18	48.17	11.82	15.90	48.92
K_L_	0.004943	0.001680	0.003078	0.004305	0.007024	0.003202
R_L_	0.1748	0.3840	0.2538	0.1956	0.1297	0.2465
R^2^	0.9908	0.9913	0.9957	0.9817	0.9858	0.9888
SSE	1.145	4.490	6.007	1.903	3.491	17.52
*χ* ^2^	0.1635	0.9005	2.157	1.139	1.813	1.824
RMSE	0.3384	0.6701	0.7751	0.4362	0.5908	1.324

K_F_ ((mg/g)·(L/mg)^1/n^), q (mg/g), Q (mg/g), K_L_ (L/mg).

**Table 5:** The parameters of the pseudo-first-order and pseudo-second-order kinetic models of ABk 1 adsorption on CLIN and NaP1.

	CLIN		NaP1	
	25 mg/L	250 mg/L	25 mg/L	250 mg/L
q_e_	2.51	6.3	1.46	5.12
Pseudo-first-order model				
q_e1_	1.2159	2.6797	0.9408	3.0826
k_1_	0.0044	0.0042	0.0017	0.0042
R^2^	0.8820	0.8270	0.8461	0.9113
Pseudo-second-order model				
q_e2_	2.5484	6.3573	1.4384	5.2356
k_2_	0.0457	0.0568	0.0157	0.0277
R^2^	0.9997	0.9999	0.9855	0.9999

q_e_ (mg/g), q_e1_ (mg/g), k_1_ (1/min), q_e2_ (mg/g), k_2_ (g/mg min).

**Table 6:** The isotherm models and error functions used in the study.

Isotherm Models				
Isotherm	Nonlinear Form	Linear Form	Parameters	Ref.
Freundlich	$q=K_{F}\cdot {C_{eq}}^{1/n}$	logq=logKF·1nlogCeq	K_F_—Freundlich constant associated with the system’s adsorption capacity and intensity, expressed in units of mg/g(L/mg)^1/nF^. 1/n—Represents the adsorption favorability and is a dimensionless parameter.	[54]
Langmuir	$q=\frac{q_{max}K_{L}C_{eq}}{1+K_{L}C_{eq}}$	$\frac{C_{eq}}{q}=\frac{C_{eq}}{q_{max}}+\frac{1}{K_{L}q_{max}}$	q_max_—The maximum capacity of adsorption, measured in mg/g. K_L_—The Langmuir constant, reflecting the binding site affinity and the adsorption energy, with units of L/mg.	[55]
Error functions				
Abbreviation	Definition/expression	Parameters		Ref.
SSE	$\sum_{i=1}^{n}{\left(q_{e,cal}-q_{e,exp}\right)}_{i}^{2}$	q_e,cal_—The adsorption capacity value obtained from calculations. q_e,exp_—The adsorption capacity value measured experimentally. n—The total number of data points in the experimental observations.		[53]
RMSE	$\sqrt{\frac{1}{n}\sum_{i=1}^{n}{\left(q_{e,exp}-q_{e,cal}\right)}_{i}^{2}}$			
*χ* ^2^	$\sum_{i=1}^{n}\frac{{\left(q_{e,exp}-q_{e,cal}\right)}^{2}}{q_{e,cal}}$			

**Table 7:** Kinetics equations.

Kinetic Model	Equation	Nonlinear Form	Description of Kinetic Parameters	Ref.
Pseudo-first-order	$\frac{dq_{t}}{d_{t}}=k_{1}\left(q_{e}-q_{t}\right)$	$q_{t}=q_{e}\left(1-e^{-k_{1}t}\right)$	q_t_ (mg/g)—the amount of ABk 1 adsorbed at any specific time (min). q_e_ (mg/g)—the amount of ABk 1 adsorbed at equilibrium. k_1_ (1/min)—the adsorption rate constant, potentially influenced by the initial concentration of the solute. k_2_ (g/(mg·min))—the adsorption rate constant for the pseudo-second-order model. h (1/min)—the initial adsorption rate.	[3]
Pseudo-second-order	$\frac{dq_{t}}{d_{t}}=k_{2}{\left(q_{e}-q_{t}\right)}^{2}$	$q_{t}=\frac{k_{2}q_{e}^{2}t}{1+k_{2}q_{e}t}\;\;h=k_{2}q_{e}^{2}$		

## Data Availability

Dataset available on request from the authors.
